# Applications of Optical Fiber in Label-Free Biosensors and Bioimaging: A Review

**DOI:** 10.3390/bios13010064

**Published:** 2022-12-30

**Authors:** Baocheng Li, Ruochong Zhang, Renzhe Bi, Malini Olivo

**Affiliations:** Translational Biophotonics Laboratory, Institute of Bioengineering and Bioimaging, Agency for Science, Technology and Research (A*STAR), Singapore 138667, Singapore

**Keywords:** biosensor, bioimaging optical fiber biosensor, surface plasmon resonance, Mach–Zehnder interferometer, fiber Bragg grating, photoacoustic imaging

## Abstract

Biosensing and bioimaging are essential in understanding biological and pathological processes in a living system, for example, in detecting and understanding certain diseases. Optical fiber has made remarkable contributions to the biosensing and bioimaging areas due to its unique advantages of compact size, immunity to electromagnetic interference, biocompatibility, fast response, etc. This review paper will present an overview of seven common types of optical fiber biosensors and optical fiber-based ultrasound detection in photoacoustic imaging (PAI) and the applications of these technologies in biosensing and bioimaging areas. Of course, there are many types of optical fiber biosensors. Still, this paper will review the most common ones: optical fiber grating, surface plasmon resonance, Sagnac interferometer, Mach–Zehnder interferometer, Michelson interferometer, Fabry–Perot Interferometer, lossy mode resonance, and surface-enhanced Raman scattering. Furthermore, different optical fiber techniques for detecting ultrasound in PAI are summarized. Finally, the main challenges and future development direction are briefly discussed.

## 1. Introduction

With the advancement of optical fiber technologies and the unique advantages such as small size, lightweight, immune to electromagnetic interference, excellent light transmission, and low cost, optical fiber has demonstrated its importance in various applications such as communications, laser systems, sensing, and imaging. For example, Charles Kao proposed that a low-loss optical fiber could be achieved using high-purity glass fibers [1], and the loss has reduced dramatically to 0.2 dB/km in 1979 [2]. Furthermore, the low-loss optical fiber exhibits outstanding potential in biology applications, especially biosensors and bioimaging.

Biosensors often require labels to facilitate biomolecular measurements due to the difficulties in detecting based on the intrinsic physical properties of the analytes only. However, the labeled biosensor techniques are costly and time-consuming in multistep detections. Label-free biosensors have attracted much research attention and progressed excessively lately because they are low-cost and possess the potential for rapid and real-time sensing of target analytes. Optical fiber biosensors are great alternatives to the traditional methods for label-free biomolecular measurements because of high sensitivity, selective and multi-parameter sensing. Based on the sensing mechanisms, the most promising optical fiber biosensors can be classified as optical fiber grating [3], surface plasmon resonance (SPR) [4], Mach–Zehnder interferometer (MZI) [5], Michelson interferometer (MI) [6], Fabry–Perot interferometer (FPI) [7], Sagnac interferometer [8], lossy mode resonance (LMR) [9], and surface-enhanced Raman scattering (SERS) [10], etc. The optical fiber biosensors produce signals that are proportional to the concentration of analytes, and the received signals are analyzed through different methods such as tracking of wavelength shifts [11], intensity fluctuation [12], phase shift [13], and frequency shift [14]. Accurate sensing of low-concentration analytes could assist in early stage illnesses diagnosis [12]. Moreover, optical fiber biosensors have demonstrated promising detection abilities in DNA, glucose, protein, living cells, antibiotics, viruses, bacteria toxins, tumor cells, etc. The miniaturized and compacted biosensors are desired in biomedical, biochemistry, and clinical trial. Furthermore, optical fiber biosensors are highly compatible with optoelectronics devices with almost the same working wavelength range. Optical fibers have to be functionalized for the detection of desired biomolecules. Different functionalization techniques were deployed in optical fiber biosensors, for example, in physisorption [15,16], using organosilanes to bind biomolecules such as (3-Aminopropyl)triethoxysilane (APTES) [17,18], Poly-L-lysine (PLL) [19,20], etc. To improve the biosensors’ sensitivities, special optical fiber structures have been proposed, for instance, etched optical fiber, side-polished optical fiber, infiltration of high sensitivity liquids or liquid crystals in photonic crystal fiber (PCF), tapered optical fiber, etc. [21,22,23,24,25,26]. These highly sensitive optical fiber biosensors are designed with in-line structure, strengthening the benefits in biology applications and clinical trials. A brief overview of these special optical fiber structures will be introduced in this review.

The optical fiber could be utilized in photoacoustic imaging (PAI) applications other than biosensors. The photoacoustic phenomenon involves interactions of light and sound waves where a pulsed laser is used to stimulate tissue and deposit heat which leads to thermal expansion and pressure wave formation. Piezoelectric ultrasound transducers are widely used to detect the generated pressure, normally centered at several MHz with 50–100% bandwidth. However, the detection method has several limitations, including limited bandwidth and sensitivity, contact measurement with coupling electromagnetic interference, bulky size, etc. Optical-fiber-based ultrasound detections, have drawn lots of attention since the early 20 s [27]. Which might be superior in PAI owing to the wideband, high sensitivity, electromagnetic interference immunity, small size, and flexibility, etc. Many types of optical fiber-based ultrasound detectors for PAI applications have been reviewed [28,29,30]. Herein, we will update the progress in recent five years.

This paper reviews recent developments and the state-of-the-art technologies of label-free optical fiber biosensors and PAI based on optical fiber detection. This article contains two main parts. The first part discusses different light guiding mechanisms, geometrical designs, working principles, and recent applications for optical fiber biosensors, followed by optical fiber detection in PAI applications in the second part.

## 2. Optical Fiber Grating-Based Biosensors

Optical fiber grating (OFG) is a fiber with periodical gratings inscribed on the fiber core, resulting in a periodical change of the RI of the fiber core. Based on the period of gratings, OFG could be classified as fiber Bragg grating (FBG) and long period grating (LPG). The grating period for FBG is typically a few hundred nm, while LPG has grating periods ranging from 100 μm to 1 mm. FBG has excellent multiplexing capabilities; multiples of FBGs could be inscribed on the same fiber, which opens up the potential for multi-parameter sensing [31]. FBG-based biosensors required special designs such as etched FBG and tilted FBG (TFBG) to increase interactions between the fiber core and the RI of external environment. Besides, The OFG could incorporate nanotechnology by applying/growing nanostructured coatings on the OFG surface to increase the field interaction at the molecular level. The nanostructured coatings could be designed to excite SPR, LSPR, or lossy mode resonance.

### 2.1. Fiber Bragg Grating

In FBG, a narrow band of the input signal is reflected due to the Bragg conditions while the rest of signal is transmitted. The central wavelength of the reflected signal is Bragg wavelength *λ*_B_ and it can be expressed as [32]:

(1) $$\lambda_{B}=2n_{\mathrm{eff}}\Lambda ,$$

where $n_{\mathrm{eff}}$ is the effective refractive index (RI) of the FBG core and *Λ* is the grating pitch/period. The standard FBG is insensitive to the surrounding RI as the light is confined in the fiber core. Moreover, optical fiber biosensors mainly depend on the detection of RI variations. Therefore, reducing the thickness of FBG promotes a stronger interaction between the evanescent wave field of light propagating mode and the surrounding RI.

**Figure 1:**
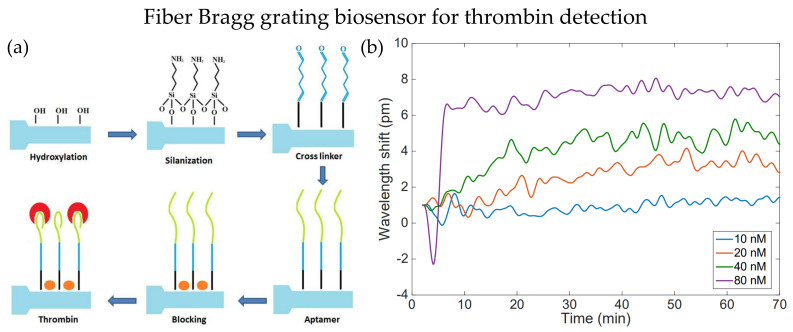
(**a**) Process of sensor probe preparation for thrombin detection. (**b**) Wavelength shift for detection of thrombin with different concentrations using EFBG, the results are observed for 70 min. Adapted with permission from Ref. [33]. Copyright 2018, MDPI.

The fiber cladding thickness can be reduced by etching method, and it is commonly used to enhance optical fiber’s RI sensitivities, especially for OFG. Etched FBG is one of the modifications that make FBG more sensitive to the surrounding RI. In 2018, Bekmurzayeva et al. proposed a wet-etched FBG biosensor to detect thrombin [33]. The FBG sensor probe was etched with hydrofluoric (HF) acid for about 27 min before functionalization. Functionalization steps are shown in Figure 1a. First, the sensor probe was cleaned with Piranha solution and became more hydrophilic, and then it was silanized with (3-Aminopropyl)triethoxysilane (APTES). Then, it is treated with thrombin aptamer and blocked on the surface with bovine serum albumin to reduce unwanted bindings. Thrombin with different concentrations within 10 nM to 80 nM could be detected by the etched FBG biosensor, as shown in Figure 1b. The measurement accuracy improved as the observation time increased, and the Bragg wavelength fluctuated within 0.3 pm. Preparation of such biosensors does not require sophisticated coating processes of additional layers on the fiber surface. Microfiber FBG is another technique that could be utilized in biosensors besides etching. In the same year, Liu et al. demonstrated inscribing FBG on microfiber with a diameter of 3.5 μm as an immunosensor for diagnosing cardiovascular disease [34]. A π-phase-shift is introduced to the FBG during inscribing processes. As a result, a narrow-linewidth notch is observed in the reflection spectrum. The notch enhanced the detection ability in small amounts of immune binding events. The common FBG fabrication method is the phase mask technique, where high photosensitivity fibers are required, and complex grating structures need expensive phase masks. Ultrafast-laser-inscription (ULI) is an alternative method for FBG fabrication, photosensitivity fibers are not required in ULI, and it can fabricate FBG with higher harmonic orders.

**Figure 2:**
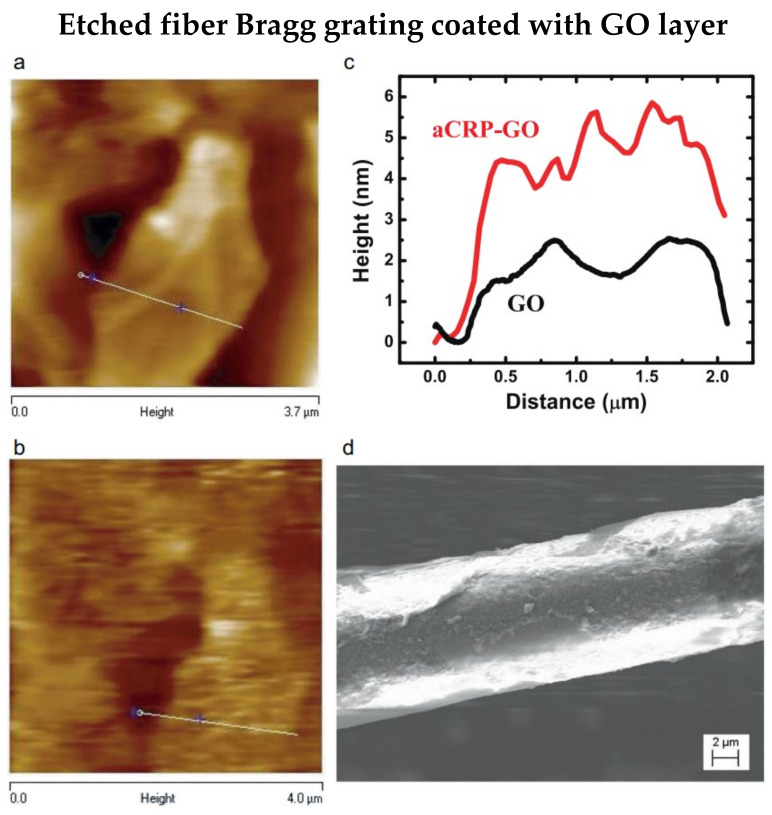
Atomic Force Microscopy image of pre (**a**) and post (**b**) functionalization of the etched FBG. (**c**) Height changes of GO layer, median height of 1.5 nm and 4.5 nm were observed pre and post functionalization of anti-CRP. (**d**) SEM image of functionalized optical fiber biosensor. Reprinted with permission from Ref. [36]. Copyright 2015, Elsevier.

In 2018, Schulze et al. fabricated an etched FBG using a femtosecond-pulsed laser to detect C-reactive protein (CRP) [35]. The nonlinear photoionization mechanisms are utilized during the inscription of gratings by femtosecond-pulsed laser, and HF acid is used for the etching process. The single-stranded DNA (ssDNA) aptamer is used for biofunctionalization and achieving a low limit of detection (LOD) of 0.82 pg/L for CRP detection. As demonstrated by Sridevi et al., an anti-CRP-graphene oxide (GO) layer was coated on the etched FBG to detect CRP [36]. Images of GO flake before and after functionalization are shown in Figure 2a,b, respectively. Additionally, the average height increased by about 3 nm after anti-CRP binding is shown in Figure 2c. Furthermore, Figure 2d illustrates the anti-CRP increases the roughness of the biosensor surface. The LOD is 0.01 mg/L, and a linear response was proved for the CRP range of 0.01 mg/L to 100 mg/L. Furthermore, the biosensor showed a high selectivity when urea, creatinine, and glucose were present in the test analytes. Besides, a layer of reduced GO can be coated on the etched FBG to detect and quantify double-stranded DNA [37]. Therefore, the proposed biosensor does not require additional functionalization; instead, the biosensor depends on the absorption of double-stranded DNA on the reduced GO surface. The LOD for double-stranded DNA is 261.87 pg/μL.

### 2.2. Tilted Fiber Bragg Grating

The gratings written on the TFBG are tilted uniformly with an angle *θ* with respect to the axial normal line of fiber. The resonance wavelength for TFBG cladding mode at particular *m*th order can be expressed as [38]:

(2) $$\lambda_{\mathrm{clad}}^{m}=\left(n_{\mathrm{eff},\mathrm{core}}+n_{\mathrm{eff},\mathrm{clad}}^{m}\right)\frac{\Lambda }{\cos\theta },$$

where $n_{\mathrm{eff},\mathrm{core}}$ and $n_{\mathrm{eff},\mathrm{clad}}^{m}$ are the effective RI of optical fiber core and *m*th order cladding mode, respectively. The cladding guides the resonance wavelength of TFBG cladding modes, and the effective RI of the cladding is affected by the cladding-surrounding interface.

**Figure 3:**
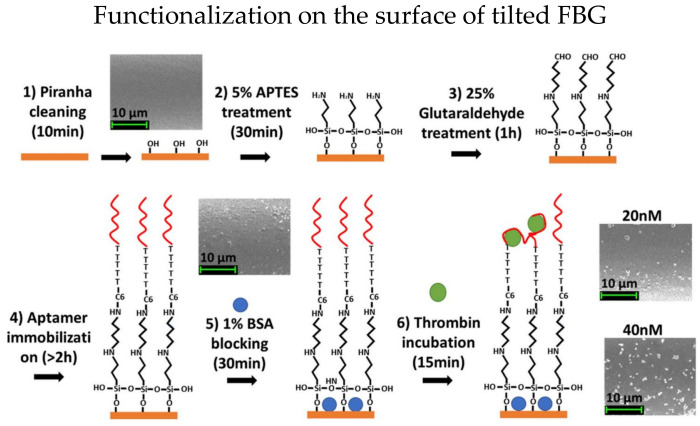
Functionalization steps of etched TFBG for thrombin detection. Reprinted with permission from Ref. [40]. Copyright 2015, Elsevier.

Etched TFBG has significantly enhanced RI sensitivities for biosensors [39]. In 2019, Sypabekova et al. functionalized etched TFBG for protein detection [40]. The RI sensitivities have been enhanced more than 18 times after etching, and the enhancement does not require any metal depositions. The fiber surface was functionalized with thrombin aptamer, and the steps are shown in Figure 3. Functionalized etched TFBG was utilized to detect thrombin with different concentrations of 2.5 to 40 nM and the best sensitivity achieved was 3.3 pm/nM. Moreover, the LOD is about 0.075 nM to 0.11 nM theoretically.

TFBG coated with a metallic layer could excite SPR and possess advantages of SPR and TFBG. The metallic coating layer has stronger electromagnetic energy, resulting in higher sensitivity to the surrounding RI. The common metallic layer used on the fiber surface is gold because of its biocompatibility and stainless property. In 2019, Chen et al. demonstrated a 50 nm-thick gold film coated on the TFBG surface to detect S-adenosyl-L-homocysteine (AdoHcy) and monitor the interaction between protein Set7 and AdoHcy in real-time [41]. The TFBG was then functionalized by protein lysine methyltransferase Set7. The LOD of AdoHcy is 1 nM. Besides, the sensor system design is combined with a microfluidic system, which could monitor the AdoHcy and Set7 interaction in real-time. The sensor system was further improved in 2021, where a 50 nm-thick gold nanofilm was coated on the TFBG for calmodulin detection [42]. The gold nanofilm surface was bonded with transient receptor potential channels to act as bio-receptors. The LOD was 0.44 nM for calmodulin detection. The microfluidic system realized the real-time monitoring of calmodulin and transient receptor potential interaction.

As another example, Lobry et al. coated a gold layer with thickness of 35 nm on TFBG for D-glucose biosensing [43]. The thin gold layer was deposited using a sputtering machine, and the polydopamine (PDA) layer was subsequently coated on the thin gold layer by the polymerization process. Then, the biosensor probe was immersed in Con A solution to link lectin protein to PDA. They reported a low LOD of 10^−7^ M and the highest sensitivity was observed in 10^−6^ to 10^−4^ M concentration range of D-glucose.

There are several narrow attenuation bands in the transmitted spectrum of a TFBG. The common method of analyzing the spectrum is tracking the wavelength shift or amplitude fluctuations of the cladding mode that has highest sensitivity. However, the common analysis method might only provide the most sensitive and reliable method some of the time. Recently, Vidal et al. deployed TFBG as an immunosensor with bare TFBG and Au-coated TFBG [44]. By tracking the lower envelope of the transmitted spectrum, the results showed the lowest LOD of 0.75 ng/mL and 0.19 ng/mL when detecting N-terminal B-type natriuretic peptide (NT-proBNP) using bare TFBG and Au-TFBG, respectively.

### 2.3. Long Period Grating

Unlike conventional FBG, LPG usually has grating periods between 100 μm to 1 mm. The coupling modes of LPG involve only the fundamental core mode and co-propagating cladding modes, which results in several attenuation dips in the transmitted spectrum. Based on the phase-matching condition, the resonance wavelengths of LPG can be expressed as [45]:

(3) $$\lambda_{\mathrm{res}}^{m}=\left(n_{\mathrm{eff},\mathrm{core}}-n_{\mathrm{eff},\mathrm{clad}}^{m}\right)\Lambda .$$

Equation (3) shows the resonance wavelength depend on the effective RI of fiber cladding, which is depending on the cladding-surrounding interface. The resonance wavelengths have red shift when the surrounding RI is decreased.

**Figure 4:**
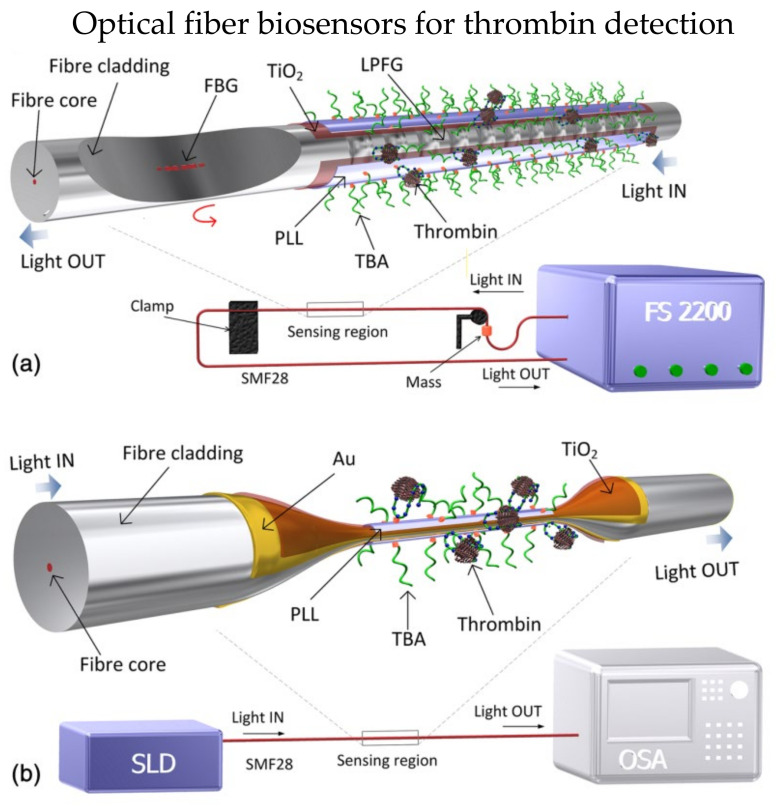
Schemes of biosensor for thrombin detection: (**a**) A layer of TiO_2_ was coated on the long period fiber grating (LPFG), an FBG was inscribed on the same fiber; (**b**) Au and TiO_2_ were coated on the etched single-mode fiber (SMF) to excite SPR. Reprinted with permission from Ref. [46]. Copyright 2016, SPIE.

In 2016, Coelho et al. presented aptamer-based optical fiber biosensors utilizing LPG and SPR for thrombin detection [46]. Two biosensors were presented in their work, as shown in Figure 4. First, a 30 nm-thick titanium dioxide layer was coated on the LPG surface to enhance the surrounding RI sensitivity, as shown in Figure 4a. The SPR biosensor was based on etched optical fiber, which comprises three coating layers, a 2 nm-thick chromium layer was coated on the fiber surface to enhance the adhesiveness of gold. Then, a 16 nm-thick gold layer was deposited on the chromium layer. Finally, a 100 nm-thick titanium dioxide layer was coated on the gold layer, as shown in Figure 4b. Then, the thrombin-binding aptamer (TBA) was immobilized on the sensor probe via poly-L-lysine (PLL). The LOD for thrombin was 10 nM for both biosensors. Although SPR biosensor has a higher RI sensitivity and figure of merit, LPG has its unique advantage with the capabilities in multiplexing.

Furthermore, in 2020, Xu et al. experimentally studied a GO-coated bare LPG to monitor the response of glucose concentrations [47]. The glucose oxidase (GOD) was immobilized on the GO layer using the chemical crosslink method, and the crosslinking reagents used are 1-ethyl-3-(3-dimethylaminopropyl) carbodiimide (EDC) and N-hydroxyl succinimide (NHS). As the proposed biosensor depends on enzyme activity and is affected by pH values, the experiment was conducted at pH = 7.0, where the enzyme activity reaches a peak. As a result, they achieved a sensitivity of 0.77 nm/(mg/mL) in glucose concentrations under 1.2 mg/mL.

Chemical etching of LPG cladding could reduce the cladding size effectively, increasing the surrounding RI sensitivity. In 2021, Esposito et al. reported an etched LPG coated with GO to detect CRP in serum [48]. Double cladding fiber was used in this biosensor where the RI of the outer cladding is higher than the inner one. The etched outer cladding achieved the mode transition phenomenon and resulted in a significant increment of RI sensitivity. The GO thin layer was deposited on the surface of etched double-cladding, which is for the covalent immobilization of the antibody (monoclonal antibody clone C5). They reported a low LOD of 0.15 ng/mL, and the detection range is 1 ng/mL to 100 μg/mL. In addition, they have reported a biosensor for detecting vitamin D using the same technique with different antibodies in the same year [49]. The biological recognition element used is 25(OH)D3. By etching the outer cladding, the RI sensitivity has significantly enhanced to about 1400 nm/RIU. The LOD for the detection of vitamin D reported was 1.0 ng/mL, and the detection range is 1–1000 ng/mL.

Tapering is another method of reducing optical fiber size to increase the RI sensitivity. In 2020, wang et al. demonstrated a tapered LPG biosensor functionalized by GO for hemoglobin detection [50]. The tapered fiber has a diameter of 110 μm coated with a GO layer via physical adsorption. The cross-linking agent APTES was used in the chemical bonding method. The GO layer adsorbs hemoglobin molecules to realize hemoglobin detection. The study reported the biosensor in detecting human hemoglobin exhibits the highest sensitivity of −2 nm/(mg/mL) in the DI water, and the LOD is 0.02 mg/mL.

Besides chemical etching and tapering to increase the RI sensitivity, LPG operating near the turn-around point (TAP) or dispersion turning point (DTP) is another effective technique to obtain higher RI sensitivity [51]. Having LPG operating around the TAP/DTP, two resonance wavelengths satisfying phase-matching conditions can be observed in the same cladding mode. It is easier for LPG to operate near the TAP if the grating period is small and the higher cladding mode is coupled with the core mode. In 2018, Liu et al. experimentally presented LPG based biosensor operating close to the TAP to detect streptavidin (SV) and immunoglobulin M (IgM) [52]. The LPG was coated with silica nanoparticles (NPs), and it is then functionalized using APTES with a silanization process to attach gold NPs to the silica NPs. Layer-by-layer (LbL) method was utilized to ensure uniform surface coverage of coating. Biotin was covalently bonded to the LPG to detect SV, and the detection sensitivity was 3.88/(ng/mm^2^) for SV concentrations 1.25 nM to 2.7 μM. Anti-IgM was bonded on the LPG surface to detect IgM, and the detection sensitivity is 11 nm/(ng/mm^2^) for human IgM concentrations of 15.6 μg/mL to 1 mg/mL. The LOD is 0.86 pg/mm^2^ and 15 pg/mm^2^ for detecting SV and IgM, respectively.

Moreover, the aforementioned techniques can be combined to maximize the RI sensitivity potential. In 2021, Dey et al. demonstrated an etched LPG biosensor operating near the TAP for immunoassay [53]. This combination has significantly enhanced the RI sensitivity to about 8751 nm/RIU [54]. The etched LPG has a diameter of 21.88 μm with a grating period of 246 μm. A layer of Eudragit L100 was deposited for the immobilization of Immunoglobulin G (IgG). The immunoassay was achieved by adopting IgG/anti-IgG interaction in human serum. They reported a low LOD of 1.06 pM.

## 3. Surface Plasmon Resonance-Based Biosensors

Surface plasmon resonance (SPR) was first discovered by Wood in 1902, where the light distribution is uneven in a diffraction grating spectrum [55]. SPR is the resonance of electron oscillations at the metal/dielectric interface stimulated by light. The SPR experiments are based on a prism system where the prism modulates the incident light before hitting the negative permittivity/dielectric interface. The negative permittivity materials for the excitation of surface plasmons are usually thin metal films such as gold (Au) and silver (Ag). The dielectric material is typically the analyte in liquids. Otto has a detailed explanation of prism-based SPR with the Otto configuration in 1968 [56]. Although the Otto configuration is based on the prism-analyte-metal film, this configuration was rather complex. Therefore, it upgraded to the Kretschmann configuration with a prism-metal film-analyte structure in 1971 [57]. The resonance conditions of SPR are highly dependent on the analyte liquid’s refractive index, and the analyte’s concentration levels are proportional to the RI variations. The RI variations of the analyte liquids result in wavelength shifts of the resonance dips. The excellent performance of SPR-based technologies has been deployed as biosensors to detect the recent COVID-19 virus and the test result requires only a few minutes [58]. The prism-based SPR has promising results in sensing applications. However, they suffered from system complexity and bulky, which is unrealistic for a cost-effective, portable biosensor. Therefore, optical fiber has been proposed to replace the prism as the light-guiding medium. A thin metal film layer (typically tens of nm) is usually coated on the optical fiber surface. The analyte can be recognized and captured by biomolecular recognition elements on the metal film surface, resulting in an increment of local RI [59]. Generally, the optical fiber SPR biosensors are designed with an all-fiber structure which is small-size, low-cost, portable, and low-loss.

SPR-based optical fiber biosensor is one of the most sensitive label-free biosensors, especially in detecting cells, proteins, antigens, drugs, and DNA [60]. Moreover, the SPR technique is often applied to other fiber designs, similarly to fiber gratings, to enhance the surrounding RI sensitivities. To increase the sensitivity further, nanomaterials were incorporated with optical fiber biosensors resulting in an optical phenomenon: localized surface plasmon (LSP). The surface plasmon excitation generates LSP in NPs that has smaller size than the wavelength of light [10]. As a result, optical fiber biosensors face challenges in detecting low-concentration and small-molecule samples. The LSP is more suitable for sensing smaller biomolecules due to the electromagnetic field’s confinement that reduces the evanescent field’s penetration depth [61].

Hollow core fibers have impactful applications in SPR-based biosensors thanks to the unique advantages such as high sensitivity, mechanically strong, and ease to manufacture [62,63,64,65,66,67,68]. In 2019, Kumar et al. demonstrated a hollow core fiber coated with gold NPs to detect cholesterol [69]. The cholesterol oxidase enzyme was used in the gold NPs coated sensor probe to enhance the sensing selectivity. The detection range of cholesterol was 10 nM–10 mM, and the LOD was 25.5 nM. Other special designs of SPR-based optical fiber biosensors such as D-shaped fiber [70,71,72,73] and tapered fiber [24,74,75] enhanced the sensitivity significantly but suffered from weaker mechanical strength.

**Figure 5:**
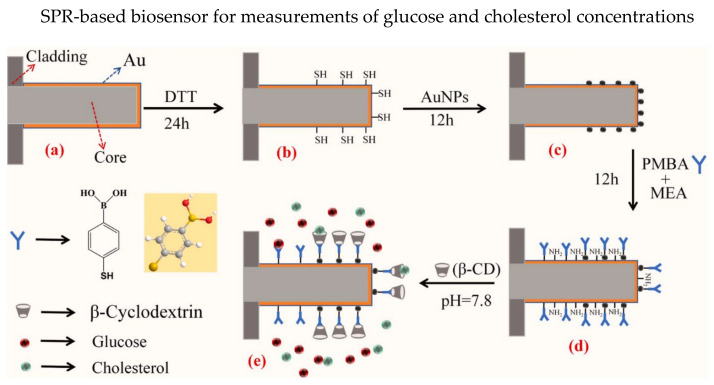
Process of sensor probe preparation for dual parameters measurements of glucose and cholesterol concentrations. (**a**) The Au coated fiber tube is immersed in dithiothreitol (DTT) for 24 h to form the free mercapto groups in (**b**). (**c**) The Au nanoparticles (AuNPs) are connected to the Au film by the mercapto groups. (**d**) After which the sensor probe is immersed in PMBA and mercaptoacetamide (MEA) mixture for 12 h and (**e**) immersed in 0.01 M β-CD solution that was prepared using PB buffer solution of pH = 7.8. Reprinted with permission from Ref. [4]. Copyright 2022, Elsevier.

Multi-parameter sensing has attracted tremendous research interest in biosensors, in which a single optical fiber sensing probe could detect multiple parameters. Different SPR-based biosensors were proposed for multi-parameter sensing, such as several sensing regions/channels with varying coatings on a single fiber [76,77,78,79], biofunctionalized with different recognition elements [4,80], etc. Generally, several SPR transmission resonance wavelengths are presented in multi-parameter sensing. In 2022, Zheng et al. presented a reflective optical fiber SPR-based biosensor that could simultaneously detect the concentrations of glucose and cholesterol [4]. The thoughtful structure design of the biosensor enables the “plug-and-play” feature. Au thin film was coated on the 600 µm plastic-clad fiber probe, and the bottom half of the Au-coated probe was modified with Au NPs, as shown in Figure 5. The sensor probe with Au NPs was subsequently deposited with P-mercaptophenylboronic acid (PMBA) and β-cyclodextrin (β-CD). The PMBA could bind with glucose molecules while the β-CD could combine with cholesterol molecules, resulting in shifts of SPR resonance wavelengths. The wavelength shifts for glucose concentrations of 0–1.7 nM and cholesterol concentrations of 0–300 nM are 11.228 nm and 18.893 nm, respectively. Moreover, low LOD of 0.00078 nM for glucose concentrations and 0.012 nM for cholesterol concentrations were observed. Besides, cross-sensitivity between PMBA and β-CD is analyzed, concluding that PMBA is not sensitive to cholesterol.

**Figure 6:**
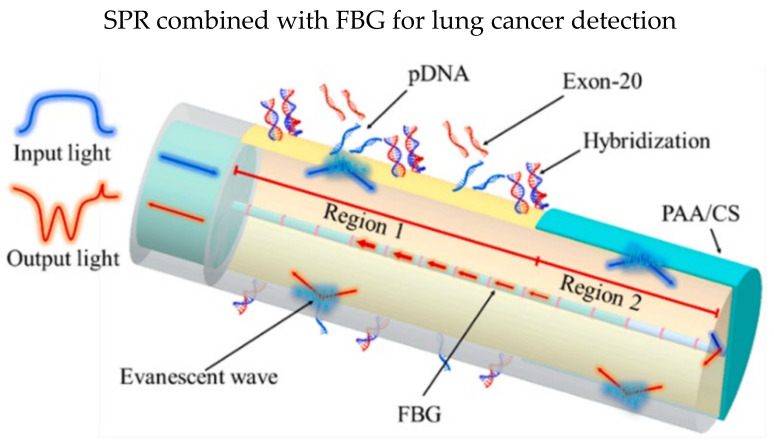
Schematic design and results of the lung cancer biosensor: The FBG region was coated with 35 nm gold film. PAA/CS (poly (acrylic acid)/chitosan) was coated on the region 2 while region 1 was functionalized with Probe DNA sequences for detection of exon-20 gene. Reprinted with permission from Ref. [81]. Copyright 2022, Elsevier.

Besides, Li et al. demonstrated an SPR biosensor that can detect three parameters simultaneously in a single fiber: epidermal growth factor receptor (EGFR) gene, temperature, and temperature [81]. Optical fiber biosensors are vulnerable to temperature and pH. Two SPR in an FBG was designed in the sensor probe, as illustrated in Figure 6, and the FBG was coated with 35 nm gold film before functionalization. Probe DNA sequences (pDNA) were immobilized in region 1 to detect exon-20, while PAA/CS was coated in region 2 using LbL technique for monitoring pH values. They reported a low LOD of the EGFR gene of 13.5 nM and a sensitivity of 0.04 nm/nM when detecting exon-20. The multi-parameter optical fiber biosensor has fulfilled the needs for clinical analysis where temperature and pH values affect the sensing accuracies.

## 4. Sagnac Interferometer-Based Biosensors

The Sagnac interference happens when the light beams from the two directions around the Sagnac loop are combined, and the two light beams are different in phase and optical path length [82]. The propagation speeds of the two light beams in the Sagnac interferometer (SI) guided mode are polarization-dependent; hence, birefringent fibers are typically used in the SI setup. SI optical fiber biosensors are relatively easier to manufacture and exhibit good stability [83].

**Figure 7:**
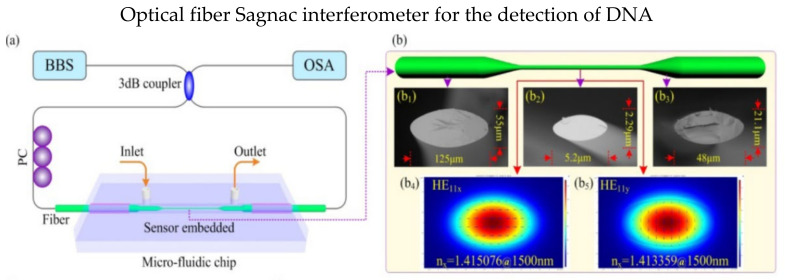
(**a**) Schematic diagram of SI DNA biosensor. (**b**) Profile images of high birefringence elliptical microfiber and the transverse electric field amplitude distributions. (**b1**) The normal size of elliptical microfiber has a major axis of 125 µm, (**b2**) the waist of the microfiber is about 5.2 µm, (**b3**) the transition regions have a major axis of 48 µm. Transverse electric field amplitude distributions of (**b4**) x−polarized mode and (**b5**) y−polarized mode. Reprinted with permission from Ref. [84]. Copyright 2017, Optical Society of America.

Specialty fibers are often deployed in the SI to increase sensitivity and biocompatibility. In 2017, Gao et al. presented a SI DNA biosensor using high birefringence elliptical microfiber as a sensing point, as shown in Figure 7a, and the waist of microfiber is only 5.2 μm [84]. Profile images of the sensor are shown in Figure 7b; the microfiber was spliced with SMF on both ends. The detection limit for the target ssDNA concentration is 75 pM. Another optical DNA sensor was proposed in 2018 by Song et al. [85]. The sensor is based on a microfluidic system within the SI. The sensing fiber is a modified side-hole fiber corroded by hydrofluoric acid. The experiment results showed a shift in wavelengths of 38.142 nm after 20 min. In 2018, Li et al. utilized the exposed core microstructure optical fiber to detect streptavidin biomolecules. Biotin was immobilized in the fiber core to capture the streptavidin biomolecules. The proposed fiber has noncircular symmetry and could access to the evanescent field directly. Moreover, it has a high RI sensitivity of −3137 nm/RIU [8].

Modified PCF could be utilized in SI as a sensing point. Modifications are to create the asymmetry in PCF to increase birefringence. In 2017, An et al. demonstrated a short PCF with different lattice pitches in SI to detect glucose [86]. The results showed an RI sensitivity of 22,130 nm/RIU and a glucose LOD of 0.76 mg/dL. In 2020, Mollah et al. proposed an optical fiber biosensor for detecting cancer cells using a special design of PCF in a Sagnac loop [87]. The sample cell is selectively filled into the elliptical air hole of the PCF. The elliptical air hole introduces asymmetry that results in a significant birefringence effect. The shifts of transmission dip due to the difference in RI between normal and cancerous cells. The results revealed the potential to detect breast cancer, blood cancer, and adrenal gland cancer.

**Figure 8:**
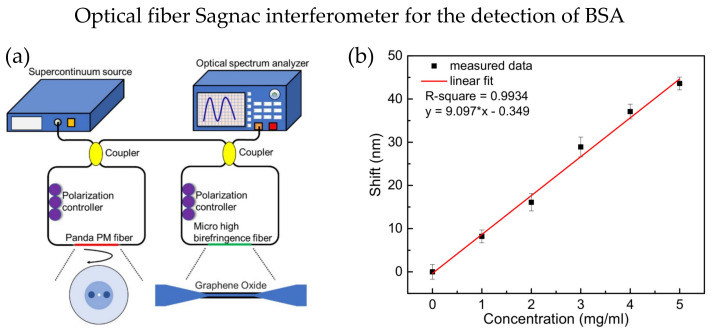
(**a**) Experimental setup of cascaded SI sensor, the micro high birefringence fiber has a diameter of 6 µm and coated with graphene oxide. (**b**) The wavelength shift of the cascaded SI sensor with difference concentrations of BSA is linear with sensitivity around 9.097 nm/(mg × mL^−1^). Adapted with permission from Ref. [88]. Copyright 2018, MDPI.

A multiple of SIs could be cascaded to increase the sensitivity, and the individual interference spectrum of each SIs is superimposed together, forming a new spectrum with the Vernier effect. Two SIs were successfully cascaded to increase the RI sensitivity by more than five times in 2018 [88]. One SI uses Panda polarization-maintaining (PM) fiber, while the other uses micro high birefringence fiber, as shown in Figure 8a. GO is coated on the micro high birefringence fiber to absorb the bovine serum albumin (BSA) molecules. The smaller the microfiber diameter, the higher the sensitivity will be. A sensitivity of 9.097 nm/(mg × mL^−1^) for BSA detection was achieved, demonstrating good biocompatibility and a large specific surface area, as shown in Figure 8b. In 2021, Zhao et al. proposed a sucrose sensor using cascaded SI with no-core fiber. The study proved that cascaded SI has increased the sucrose concentration sensitivity by 4.66 times and achieved a sensitivity of −13.84 nm/M [89].

## 5. Michelson Interferometer-Based Biosensors

**Figure 9:**
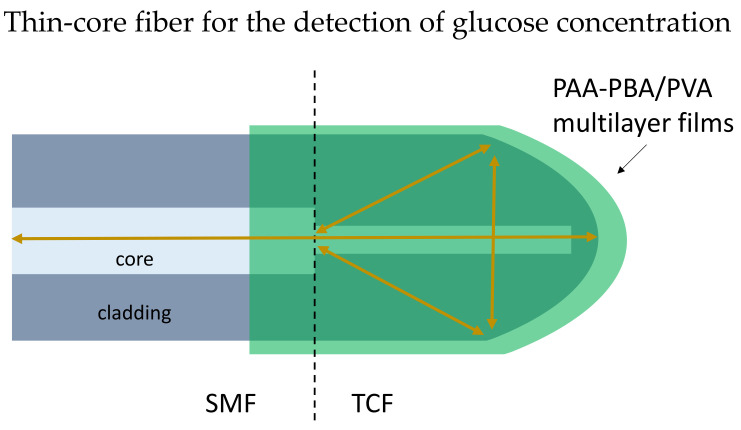
Schematic diagram of the thin-core fiber (TCF) coated with PAA-PBA/PVA films, and the TCF has a rounded tip. A SMF is spliced with the TCF.

Michelson Interferometer (MI) based biosensor has a reflecting surface at the sensor probe ends. The performance of MI fiber is observed through the reflection spectrum [90]. The MI formed when the two reflected light beams from two arms recombined in the coupler. The compact design of the MI structure biosensor contains a sensor probe that can easily be placed into a measurand solution for point measurements. In 2019, Wang et al. presented a reflection-based biosensor to detect glucose using a thin-core optical fiber [91]. The thin-core fiber tip was modified into a rounded shaped as the reflecting surface, and phenylboronic-acid-derivatized poly (acrylic acid) (PAA-PBA)/poly (vinyl alcohol) (PVA) was coated on the fiber LbL as shown in Figure 9. Experimental results proved the shift of resonance dip is linearly related to the logarithm of glucose concentration in the range of 10 μM–10 mM. The study shows the potential of application in insulin delivery systems.

As another example, Wysokinski et al. proposed an MI-based biosensor using a dual-core fiber to detect protein antigens [92]. One of the fiber cores was spliced with polarization maintaining fiber with fixed length to introduce precise arm length difference, while the other core was functionalized by immobilization of IgG on the fiber end using cross-linking reagents. An anti-IgG solution was tested using the sensor probe, the results showed a 0.6 nm shift in wavelengths when the analyte’s concentration was 160 μg/mL.

**Figure 10:**
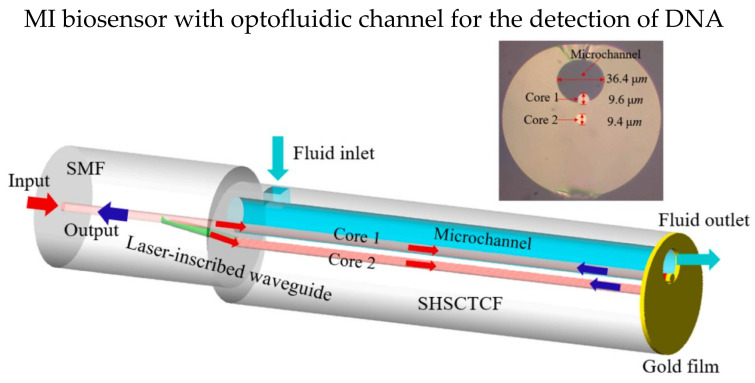
Schematic diagram of optofluidic MI biosensor. One of the cores of the twin-core fiber was exposed to the microchannel as shown in the inset. Reprinted with permission from Ref. [94]. Copyright 2022, Elsevier.

The optofluidic system can incorporate with MI design, which only requires a small amount of detection solution due to the microchannel. In 2016, Gao et al. theoretically and experimentally studied an MI-based fiber optofluidic biosensor using modified PCF to detect DNA hybridization and methylation [93]. A layer of gold film was coated on the tip of the sensor probe as the reflective surface. Part of the PCF was intentionally fused to collapse for the microchannel, and the microchannel for optofluidic was fabricated using femtosecond laser micromachining. The microchannel was coated with a layer of PLL first, then ssDNA was immobilized on the PLL layer. The LOM reported is 5 nM. In 2022, Hu et al. demonstrated an optofluidic MI biosensor that has LOD of 1 μM in detecting specify DNA solution, and only a small volume of test solution (tens of nL) is required [94]. The sensor probe of single hole suspended core twin-core fiber (SHSCTCF) end was coated with 100 nm thick gold film, and one of the cores was exposed to the microchannel, making it direct contact with air/test solution as shown in Figure 10. Core 1 was silanized with APTES and immobilized the carboxylated ssDNA on core 1. Moreover, the sensor has a high RI sensitivity of 1039.77 nm/RIU, and the LOD was 1.675 × 10^−5^ RIU.

The MI-based biosensor sensor probe is easy to implement, and the design is relatively easy. Another advantage is the pluggable measurement for point sensing in medical diagnostic applications.

## 6. Mach–Zehnder Interferometer-Based Biosensors

Similar to MI, the incident light is split into two beams in the fiber core and cladding of the Mach–Zehnder Interferometer (MZI). However, the two light beams are recombined through a coupler or splice point at the end of the fiber. Due to the variations of effective RI of fiber cladding, the optical paths are different for the two beams, and the difference is named optical path difference (OPD). The OPF introduces interference and affects the intensity of the output spectrum, which can be described as:

(4) $$I=I_{\mathrm{core}}+I_{\mathrm{clad}}+2\sqrt{I_{\mathrm{core}}I_{\mathrm{clad}}}\cos\left(\frac{2\pi ∆nL}{\lambda }+\varphi_{0}\right),$$

where *I*_core_ and *I*_clad_ are intensities of the core and cladding modes, respectively, Δ*n* is the difference of two light beams’ effective RI, *L* is the length of interference, λ is the incident light’s wavelength, *φ*_0_ is the phase difference between the two light beams initially.

According to Equation (4), the intensity of output spectrum is minimum when 2π∆*nL*/*λ_m_* + *φ*_0_ = (2m + 1)π. The effective RI difference of the two beams ∆*n* is affected by the variations surrounding RI, which result in a wavelength shifts. Hence, the MZI-based fiber could realize biosensing by coating specific films that are sensitive to target analytes.

**Figure 11:**
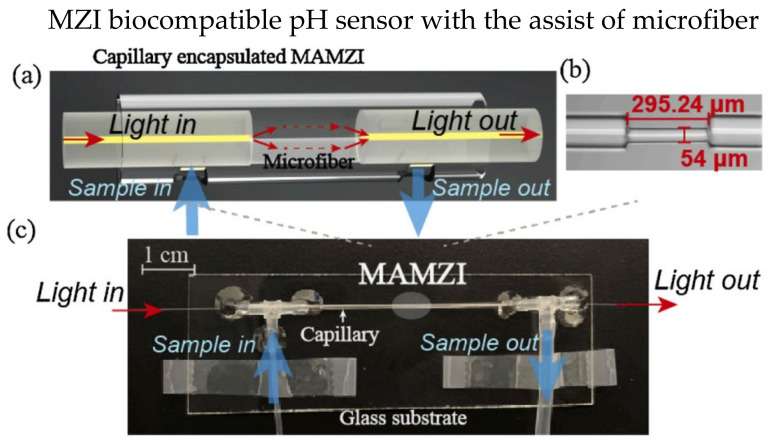
Experiment setup of the biocompatible pH sensor: (**a**) schematic diagram of the MAMZI, two SMF was spliced with a small section of microfiber to form the MZI, the microfiber was spliced with the lower half of the SMF; (**b**) A microscopic image of the MAMZI, showing a tapered diameter of 54 μm and total length of 295.24 μm; (**c**) Experimental setup with a microfluidic chip. Reprinted with permission from Ref. [95]. Copyright 2021, Elsevier.

Various designs of MZI-based biosensors have been investigated over the last decades, including microfibers [95], twin-core fiber (TCF) [96,97], thin-core fiber [98,99], etc. Microfibers are commonly used in MZI-based biosensors due to their ultra-high RI sensitivity and strong interaction of the evanescent field and surrounding environment. Generally, microfibers are manufactured using the tapering method to reduce waist diameter. A smaller diameter leads to a stronger interaction between the evanescent field and the surrounding environment, resulting in a more significant change in effective RI. In 2019, Hu et al. experimentally demonstrated an MZI-based biosensor using a tapered PCF coated with 4′-pentyl-biphenyl-4-carboxylic acid (PBA)-doped 4-cyano-4′-pentylbiphenyl (5CB) to detect penicillinase [100]. The liquid crystal is pH-sensitive, where the concentrations of H+ changes the orientations of liquid crystal molecules. The variations of liquid crystal molecules’ orientations result in changes in RI. In 2021, Wang et al. proposed a biocompatible pH sensor using a microfiber-assisted MZI (MAMZI); the sensor was complementary-DNA-enhanced [95]. The sensor was prepared by splicing two single-mode fibers (SMF) with a 295.24 μm microfiber at the bottom half of the fiber, as illustrated in Figure 11a. The microfiber was tapered to a diameter of 54 μm, as shown in Figure 11b. The microfluidic chip was incorporated with the sensor design to allow the injection of the mixed solution into the surrounding environment of the fiber, as shown in Figure 11c. The experimental results showed the best sensitivity of 480 nm/pH when detecting a pH range of 4.98–7.4. Moreover, the resolution is as low as 0.042 pH.

To enhance the sensitivity of microfibers further, DTP has been studied extensively. The sensitivity of microfibers goes to infinity when the microfiber’s diameter matches the DTP. In 2020, Xia et al. demonstrated a microfiber MZI operating around the DTP for RI sensing. The reported results reveal a RI sensitivity of 24,209 nm/RIU at RI near 1.332, with a tapered waist diameter of 4.8 μm [101]. They extended the study to enhance the RI sensitivity further and demonstrated a microfiber MZI for RI sensing theoretically and experimentally; the best sensitivity achieved was 44,271 nm/RIU in the RI range of 1.3364–1.3379 [102]. The microfiber has a tapered waist diameter of ~3.5 μm, and the output showed a DTP interference spectrum. The studies showed that the tapered waist diameter of microfiber in MZI relates to the RI sensitivity [103,104].

**Figure 12:**
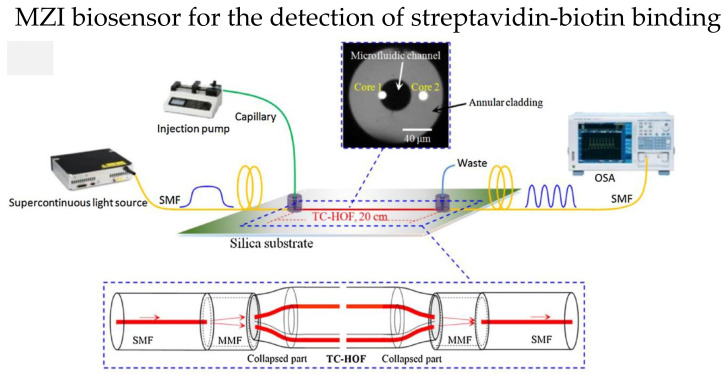
Experiment setup of biosensor to detect streptavidin-biotin binding. The core 1 of TCF serves as sensing arm while core 2 is the reference arm. The central hole of the TCF is the microfluidic channel. Reprinted with permission from Ref. [97]. Copyright 2018, Elsevier.

The two independent cores in TCF could be utilized in MZI, one of the cores as the reference arm, while the other is modified as the sensing arm. In 2018, Yang et al. reported an MZI-based streptavidin-biotin binding sensor using a TCF [97]. The TCF ends were spliced with a short multimode fiber, then spliced with an SMF, as shown in Figure 12. Core 1 in the microfluidic channel is the sensing arm, while core 2 is suspended in the cladding, which serves as the reference arm. First, the microfluidic channel surface was silanized with 3-Mercaptopropyl trimethoxysilane; then, covalent bond technique was used to immobilize thestreptavidin on the sensor surface. The RI sensitivity of 2577 nm/RIU and the sensitivity of 16.9 nm/(mg/mL) in detecting biotin with a LOD of 3 μg/mL were reported.

## 7. Fabry–Perot Interferometer-Based Biosensors

The typical case of a Fabry–Perot Interferometer (FPI) has two reflecting mirrors spaced apart by a length of l. The interference occurs when the light propagates back and forth between the two mirrors, inducing a path difference. The resonance wavelength is closely related to the surrounding environment’s effective RI and the cavity length [105], making FPI-based optical fiber sensors suitable for biosensing applications.

**Figure 13:**
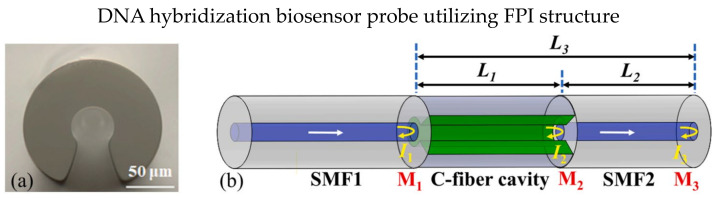
(**a**) Cross-section microscopic image of C-type fiber; (**b**) Schematic design of the biosensor, both ends of the C-type fiber are spliced with SMF and the C-type fiber serves as micro cavity. Reprinted with permission from Ref. [108]. Copyright 2022, Elsevier.

C-type fiber is frequently used in optical fiber FPI biosensor applications due to the special fiber design of C-shape, which allows analytes to fill in the fiber as a microfluidic channel, as shown in Figure 13a [106,107]. In 2019, Xie et al. presented an FPI-based biosensor using cascaded C-type fiber [106]. The two C-type fibers showed good multiplexing capability with minimal crosstalk. The biotin-streptavidin binding mechanism utilized the sensor cavity. They reported that the cascaded C-type fiber reduced the false positive measurements significantly. In 2020, Li et al. demonstrated a quantitative polymerase chain reaction (qPCR) using an all-optical system using C-type fiber [107]. The FPI cavity is constructed using C-type fiber to monitor the temperature in real time, and the biosensor is highly sensitive to fluorescence during the qPCR process. The design was improved in 2022; Li et al. proposed a DNA hybridization biosensor probe using C-type fiber with the Vernier effect [108]. The fiber structure is SMF-C-type-SMF, in which C-type fiber act as an FPI cavity, as shown in Figure 13b. The Vernier effect in the FPI cavity occurred in the cavity’s solid and air parts, exhibiting a RI sensitivity of 10,791.12 nm/RIU. The biosensor probe was functionalized by APTES and immobilizing of pDNA on the SMF surface, and the LOD was 1 μM complementary DNA. The direct interaction of analytes and fiber in C-type fiber exhibits high RI sensitivity. Still, the optical loss is expected to be increased due to the involvement of liquid analytes in the optical path.

In 2016, Liu et al. proposed an optical fiber FPI using a small section of hollow-core PCF spliced with SMFs to detect microorganism growth [109]. The experimental results showed a −138 dB/RIU sensitivity for yeast growth detection with low-temperature cross-sensitivity. The RI changes were detected by the sensor corresponding to the microorganism growth. The highly sensitive sensor design exhibits potential in biosensing applications. Moreover, their group demonstrated the same fiber structure design to serve as an immunosensor in the same year [110]. The biosensor probe was silanized with APTES then the covalent binding method was used to immobilize the anti-rabbit IgG. The experimental results showed a 190 pm wavelength increment and a 2.15 dB fringe contrast decrease. The biosensor has a compact structure, and the probe design exhibits a point-of-care feature.

In 2018, Wu et al. demonstrated an optical fiber biosensor for detecting hydrogel-based bio-toxin, and no splicing of fibers is required [111]. The FPI cavity is formed by one of the polished fiber ends and a vertical glass slide, and the hydrogel is filled in the cavity. The disulfide-cross-linked polyacrylamide (PAAm) hydrogel was cleaved by a dithiothreitol (DTT) solution. The hydrogel was degraded during this process, resulting in an effective RI change. Additionally, can be observed through the wavelength shift of the spectrum. The experimental results demonstrated a fast DTT detection. A DTT with 50 μM concentration could be detected in lesser than 36 min. In 2020, Cano-Velázquez et al. established an optical fiber FPI immunosensor using an SMF with a semi-spherical cap on the fiber end [112]. The end cap was formed by dipping the fiber end into polydimethylsiloxane (PDMS) and placed in the oven to solidify the PDMS cap, and the cap formed the FPI cavity. The PDMS cap was functionalized with bioactive lipid antigen. As a result, the biosensor could detect Sera containing different antibodies through the antigen-antibody interaction. Moreover, the dip-coating method is much easier than other coating methods.

Biosensors based on FPI require a concise length of fiber only, which can be designed as a sensor probe for point-of-care measurement. Moreover, FPI using hollow-core fiber or C-type fiber has microcavity, which can be utilized as a microfluidic channel to reduce the number of analytes required. Since cascading of micro-cavities are possible, multi-parameter sensing could be realized through cascading micro-cavities that are functionalized for different target analytes [113].

## 8. Lossy Mode Resonance-Based Biosensors

Unlike SPR, lossy mode resonance (LMR) happens when the thin-film permittivity (real part) is positive, and the magnitude is higher than the imaginary part and the surrounding material [114,115]. It is an optical resonance phenomenon caused by the coupling between a lossy mode of semiconductor thin film and a waveguide mode. The LMR-based sensor exhibits multiple resonance bands without geometry modifications to the optical fiber [116]. Moreover, it can be generated by coating thin films such as polymers and ceramics. On the other hand, various optical fiber structures were proposed to increase the RI sensitivities, such as tapered fibers [117], D-shape fiber [118,119], cladding-etched fiber [120], etc.

**Figure 14:**
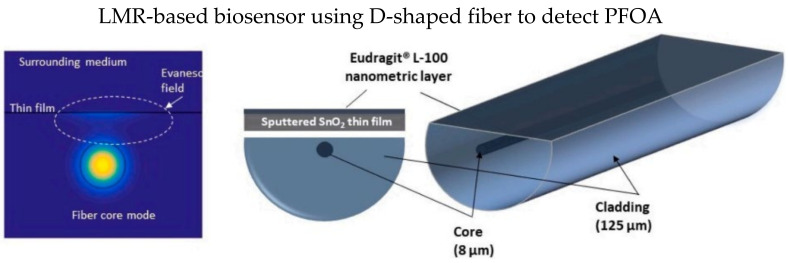
Schematic diagram of D-shape fiber coated with a thin-film layer tin dioxide (SnO_2_), which was deposited by a DC sputter machine. Reprinted with permission from Ref. [121]. Copyright 2021, Elsevier.

D-shape fiber has a polished flat surface, as shown in Figure 14, which could be fully utilized to coat a thin film layer. In 2021, Moro et al. proposed an LMR-based D-type SMF coated with a thin-film layer of tin dioxide (SnO_2_) for the detection of perfluorooctanoic acid (PFOA), as illustrated in Figure 14 [121]. SnO_2_ thin-film significantly enhances the RI sensitivity of LMR-based optical fiber sensors [122]. Delipidated human serum albumin (hSA) was covalently immobilized on the tin dioxide thin film to recognize the PFOA in a test analyte. They reported the LMR wavelength shift due to the hSA/PFOA interaction could be modeled using the Langmuir adsorption isotherm.

Biosensors that operate in multiple domains, such as optical and electrochemical, are more potent than a single domain. In 2020, Smietana et al. proposed an LMR-based optical fiber biosensor coated with indium tin oxide (ITO) for biotin-avidin detection [123]. The biosensor was interrogated electrochemically and operated in the optical domain as well. The ITO film has electrical properties that make the fiber a working electrode, and experimental results showed that LMR’s resonance conditions depend on the surrounding environment RI and the potential applied electrode. To functionalize the LMR sensor for biosensing application, the LMR part was silanized with APTES, and then the biotin was covalently bonded to the LMR surface. They observed the LMR wavelength shift was increased with increasing avidin concentration. However, the current was decreased in the electrochemical domain. In 2022, Burnat et al. coated a layer of fluorine-doped tin oxide (FTO) on optical fiber LMR as a biosensor that works in the optical and electrochemical domain [124]. The performance of the FTO-coated LMR was analyzed in both optical and electrochemical domains. The sensitivity was 450 nm/RIU for RI between 1.33–1.40; moreover, the biosensor was functionalized with biotin as the recognition element. Optical fiber LMR coated with ITO thin-film has been studied intensively, but FTO thin-film could be a promising alternative for ITO thin-film for dual domain sensor applications. Furthermore, the resonance conditions for FTO-coated LMR are greatly affected by the applied potential in a wide range.

## 9. Surface-Enhanced Raman Scattering-Based Biosensors

Surface-enhanced Raman scattering (SERS) is a popular technique for sensing molecules in trace amounts in biosensing applications. The SERS enhances excitation and inelastic scattering processes to acquire the fingerprint information of analyte molecules and potential single-molecule sensitivity [125]. In SERS, substrate materials such as GO [126] metal oxides [127] are often used to enhance the Raman spectrum intensity. The substrates could be integrated on different parts of optical fiber, such as fiber ends, side surface of fiber, etc.

**Figure 15:**
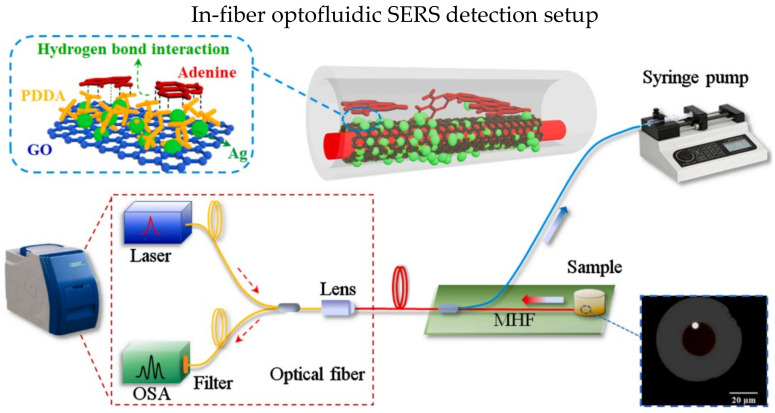
Schematic diagram of SERS-based optical fiber biosensor with optofluidic. Reprinted with permission from Ref. [126]. Copyright 2021, Elsevier.

Microstructured optical fibers have porous structure which offers larger interaction area between target analyte and the light propagating in the fiber. Moreover, the air holes in microstructured optical fibers could utilize as microfluidic channel [128,129]. In 2021, Gao et al. demonstrated an in-fiber optofluidic SERS sensor for the adenine in DNA [126]. The experiment setup is shown in Figure 15; the hollow core fiber has a suspended core within the air hole. One end of the hollow core fiber was coated with gold film as reflecting layer, while the other end was coupled with Raman probe. The suspended core was injected with piranha solution, and then the surface was functionalized with Ag NPs using APTES and ethanol. The LOD is 10^−14^ M for the detection of adenine.

**Figure 16:**
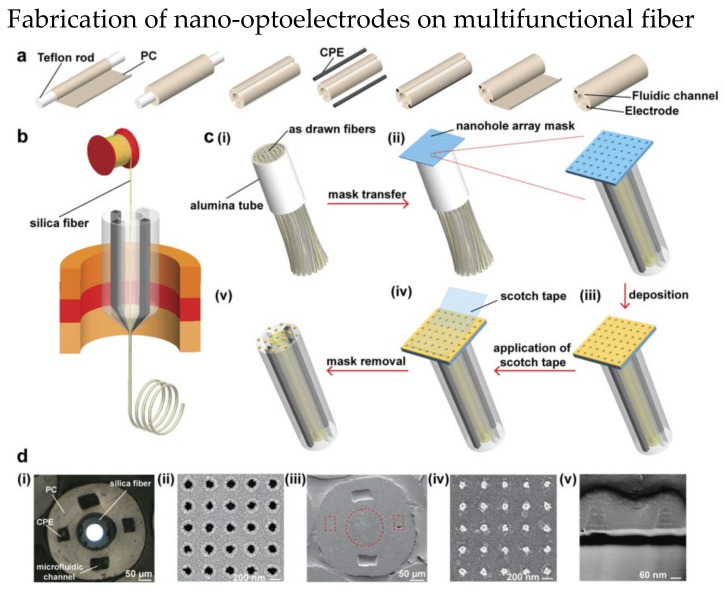
Fabrication steps of nano-optoelectrodes on multifunctional fiber probe. (**a**) Fabrication of preform with two conductive polymer electrodes (CPE) and two optofluidic channel. (**b**) A silica fiber was inserted into the hollow core of the preform before the fiber drawing process. (**c**) The process of combining nano−optoelectrodes on the fiber tips. (**i**) multiple fibers were packed together in an alumina tube and cut. (**ii**) After the top surface of fiber bundle was polished, a nanohole array mask was placed on it. (**iii**) Layers of Ti, Au, and Ag were deposited to the fiber bundle. (**iv**) A scotch tape was used to remove the nanohole array mask. (**v**) The final fiber with nanostructures. (**d**) Images of multifunctional fiber. (**i**) Optical image of the fiber bundle after polishing. SEM image of (**ii**) the nanohole array mask, (**iii**) fiber end, (**iv**) nano−optoelectrodes on the fiber end (**v**) cross−sectional of nano−optoelectrodes. Reprinted with permission from [125]. Copyright 2021, American Chemical Society.

Lab-on-fiber technology was proposed to combine the excellent performance of photonic biosensors with advantages of optical fibers [130]. The main concept is to develop an ultra-compact lab in a single optical fiber as a multifunctional sensor. The conventional nanofabrication methods including bottom-up [131,132] and top-down [133,134,135] have limited potentials in many applications. These methods can barely produce excellent accuracy and high throughput optical fiber system simultaneously. In 2021, Jiang et al. demonstrated a scalable nanofabrication process to fabricate nano-optoelectrode arrays on fiber probe [125]. The details of fabrication steps are shown in Figure 16. The fiber preform with two fluidic channels are fabricated through the steps shown in Figure 16a. Fibers drawn from Figure 16b were bundled in an alumina tube, and then a nanohole array mask was placed on the fiber end as shown in Figure 16c. Layers of Ti, Au, and Ag were then deposited on the array mask and finally the array mask was removed. Figure 16d are the SEM images of the nanohole array mask and fiber with deposited nano-optoelectrodes. The fabricated fiber has the ability of conducting RI optical sensing and SERS on the fiber tip.

## 10. Optical Fiber-Based Detection of Ultrasound in PAI

**Figure 17:**
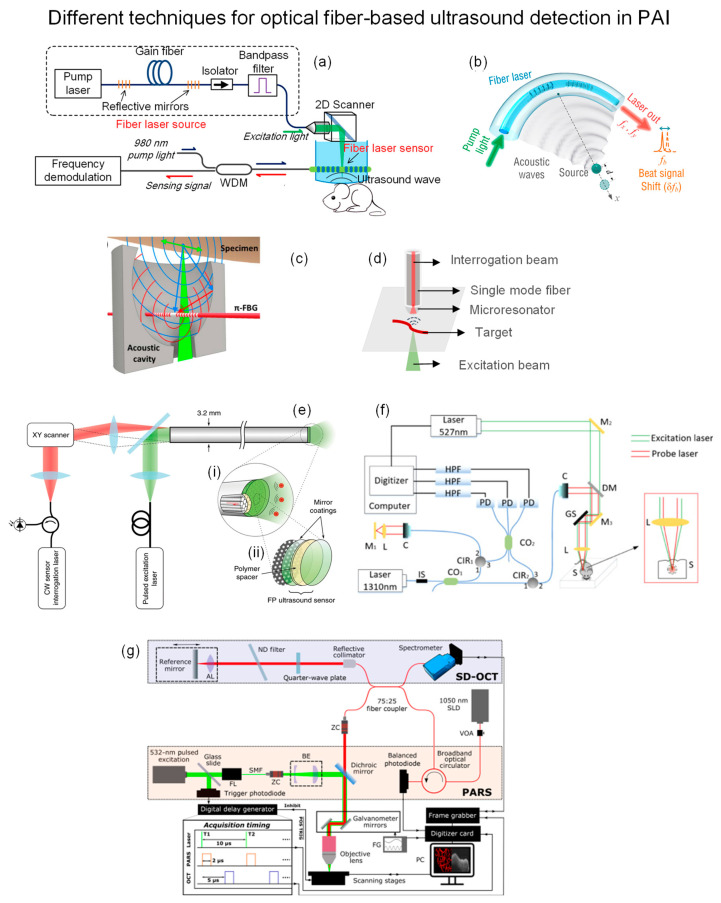
(**a**) PAM based on fiber-laser ultrasound detection. Reprinted with permission from Ref. [137]. Copyright 2021, Springer Nature. (**b**) Lens-free focused fiber-laser detector for PAT. Reprinted with permission from Ref. [138]. Copyright 2019, Optical Society of America. (**c**) π-FBG interferometer in acoustic cavity for PAM. Reprinted with permission from Ref. [139]. Copyright 2017, Optical Society of America. (**d**) Microresonator on fiber tip for ultra-sensitive acoustic detection. (**e**) Fabry-Pérot (FP) polymer film at distal tip end of fiber bundle for PAE. (**i**) The zoom-in visualization of distal end showing the optical fiber cores and FP ultrasound sensor. (**ii**) FP sensor was integrated on the distal end of fiber. Reprinted with permission from Ref. [141]. Copyright 2018, Springer Nature. (**f**) Setup of interferometric non-contact PAM. Reprinted with permission from Ref. [142]. Copyright 2019, Optical Society of America (**g**) Dual-modality non-interferometric PA remote sensing and OCT with fiber optic. Reprinted with permission from Ref. [144]. Copyright 2021, SPIE.

Optical fiber-based ultrasound detection for PAI has been demonstrated in various modalities, including microscopy (PAM), tomography (PAT), and endoscopy (PAE). Recently, Liang et al. developed a fiber-laser-based detector utilizing beat-frequency variation induced by ultrasonic waves [136,137]. The fiber laser has two wavelengths matched FBGs imprinted in series with pitch size Λ = 1053 nm. Generated acoustic wave changed the refractive index and bent the fiber, leading to birefringence change and beat-frequency shift. Figure 17a shows the demonstrative PAM setup of this fiber-laser-based acoustic detector. It was placed closely on top of the sample in a water tank. The principal axis of the fiber laser detector was aligned with the ultrasound propagation direction to optimize the collection efficiency. A 2D scanner reflected a 532 nm pulsed laser to excite the sample. The perturbed frequency was measured by a photodetector and then processed by I/Q demodulation in real time. The axial and lateral resolutions of imaging results were 48 μm and 3.3 μm, respectively. Apart from PAM, this group also demonstrated PAT application with a slight modification of the fiber laser detector by bending it at a 30 mm curvature radius to achieve lens-free focused ultrasound detection, as shown in Figure 17b [138]. Then, the focused detector was scanned around the sample, which was illuminated by an expanded 532 laser beam from the top at 0.36° step size. The image was reconstructed by 2D back projection. Axial and lateral resolutions were 500 μm and 70 μm, respectively.

Rami S. et al. embedded the π-phase-shift FBG in an acoustic cavity to make it a miniaturized photoacoustic sensor and demonstrated the PAM application [139]. The cavity with a 7.3 mm aperture focused the ultrasound on the π-FBG sensor to amplify the signal (Figure 17c). This configuration also enabled reflection mode detection that is adaptable in multitude of optical microscopes. It was demonstrated by in vivo mouse abdomen imaging using a 1520–1630 nm CW laser as an interrogation beam and a 532 nm pulsed laser as an excitation beam. The transmitted intensity was monitored by a wideband photodetector, which was altered by the acoustic wave due to resonance shift. As a result, a 3.7 µm lateral resolution was achieved.

Besides FBG, James et al. fabricated a microresonator with a high Q-factor and attached it to a normal SMF tip to achieve broadband and sensitive detection [140]. The microresonator was made of a plano-concave polymer between two mirrors to trap the interrogation beam. The simple illustration is depicted in Figure 17d. When an excitation beam induces a pressure wave, the stress will modify the cavity geometry and change the reflected optical power of the interrogation beam, which a photodetector can detect. One of its advantages is the wide angle of reception which is suitable for collecting Omni-direction PA waves. In addition, mirror reflectivity and cavity thickness are scalable for different sensitivity and bandwidth requirements. The authors fabricated and characterized a group of fiber-tip microresonators, followed by a demonstration of in vivo PAM application on a mouse ear.

Apart from the single-element detector, Rehman A. et al. developed a fiber bundle probe with a transparent Fabry-Pérot (FP) polymer sensor at its distal end, enabling forward-viewing PA endoscopy (PAE), as shown in Figure 17e [141]. It consists of 50,000 12-µm cores with a 3.2 mm outer diameter total, and each act as an ultrasound receiver. The FP sensor is transparent for 580–1250 nm but reflective for 1400–1600 nm, allowing the excitation beam to pass through while trapping the interrogation beam. The axial resolution is constant at 31 µm, and lateral resolutions range from 45–170 µm at depths 1–7 mm.

Above mentioned methods still require physical contact with the target through a coupling medium to minimize the reflection loss and attenuation of ultrasound in air, which hinders its application in open wounds, ophthalmology, and endoscopy. Yi W. et al. utilized a fiber-optic interferometer to detect PA signals by monitoring probe beam intensity [142]. The 527 nm pulsed laser was used as an excitation beam, while the 1310 nm laser was divided into reference and probe beams by a fiber coupler. The two beams passed through two fiber circulators and were detected together with noise by three photodetectors. The measured interference signals were demodulated to obtain intensity change for PA reconstruction. Figure 17f shows the setup.

Apart from interferometry, Parsin H. et al. proposed a non-interferometric remote sensing scheme for PAM in 2017, which utilized the refractive index change at the target surface in the air [143]. The same group then upgraded the free-space system to a fiber-based one and combined it with optical coherence tomography (OCT) [144], where a 1050 nm super luminescent diode (SLD) was used as PA interrogation beam and OCT light source. The 532 nm pulsed laser excited PA signal was confocused with a 1050 nm interrogation beam. A photodetector detected 75% of the returned beam, and the remaining went to the OCT spectrometer via a circulator. PA and OCT signals were acquired sequentially at each point. The entire setup and timing diagram are shown in Figure 17g.

Table 1 summarized all the PAI systems by using optical-fiber detections, as mentioned above.

## 11. Conclusions and Future Perspectives

Applications of optical fiber in biosensing and bioimaging areas have attracted much attention lately to promote a better understanding of biological and pathological processes. This review paper has extensively discussed various types of optical fiber biosensors and bioimaging, including optical fiber grating, surface plasmon resonance, Sagnac interferometer, Mach–Zehnder interferometer, Michelson interferometer, Fabry–Perot interferometer, lossy mode resonance, surface-enhanced Raman scattering, and photoacoustic imaging. Most optical fiber biosensors are label-free, and label-free biosensors are desired for low-cost, rapid, and real-time detection. Moreover, optical fiber biosensor requires a much lower dose of analytes. Table 2 summarizes the advantages and disadvantages of different types of optical fiber biosensors.

Detection of ultrasound in photoacoustic imaging is essential to capture the pressure generated by a pulsed laser. The traditional detection methods suffer from low sensitivity, limited bandwidth, etc. optical fiber-based ultrasound detectors improve the sensitivity and reduce the bulky size of the conventional sensor. Most current optical fiber biosensor research focuses on single parameter detection, and the biosensors showed a high selectivity where the test analytes contain many biomolecules. However, different biomolecules present in the test analytes might be helpful information for the test. Therefore, multi-parameter detection could be the new focus of optical fiber biosensing. Besides, lab-on-chip technology could integrate the optical fiber setup in a single chip, making the entire biosensor setup compact and easier to operate. Such integration allows biosensors to have a better performance in clinical trials. The response time of biosensors is another point that needs more attention. The biosensors depending on binding processes require longer test time. Future developments could reduce the response time, making biosensors a fast and reliable product. Besides, noninvasive glucose sensing is a hot topic and it will benefit the society if the sensitivity can be high and accurate. The light could be shined on human skin and the reflection light could be captured to analyze for the glucose information. Although the breakthrough in this technology has yet to be confirmed, researchers could focus on this aspect to develop noninvasive glucose detection that will benefit large populations.

## Figures and Tables

**Table 1:** Optical fiber based ultrasound detections in PAI. NEP, noise equivalent pressure; A, axial resolution; L: lateral resolution.

Principle	Modality	Center Frequency (MHz)	Bandwidth (MHz)	NEP (mPa/√Hz)	Directivity	Coupling	Resolutions (µm)
Fiber laser beat-frequency [137]	PAM	23.2	50	5.66	-	Water	A: 48 L: 3.3
Bent fiber laser beat-frequency [138]	PAT	22	50	5.09	Focused ± 0.13°	Water	A: 500 L: 70
π-FBG interferometry in cavity [139]	PAM	30	20	19.67	±31.3°	Gel	L: 3.7
Fiber-tip optical microresonator [140]	PAM	DC to MHz	1–40	1.6–3.3	Up to ±90°	Gel	A: 65.9 L: 94.2
Fabry-Pérot (FP) polymer-film [141]	PAE	21	34	112–282	-	Water	A: 31 L: 45–170
Fiber-optic interferometer [142]	PAM	-	-	-	-	Air	A: - L: 11
Non-interferometric refractive index [144]	PAM	-	-	-	-	Air	A: 35 L: 2.4

**Table 2:** Overview of different optical fiber biosensor sensing mechanisms.

Principle	Advantages	Disadvantages
Optical fiber grating	Low−cost High mechanical strength Cascading capability	Sensitivity relatively low
Surface plasmon resonance	High sensitivity Low sample consumption (optofluidic)	High fabrication difficulty High cost
Sagnac interferometer	Ultrahigh sensitivity	Relatively large size Temperature cross sensitivity
Mach–Zehnder interferometer	High sensitivity High mechanical strength	High fabrication difficulty Low repeatability
Michelson interferometer	Small size Probe design	Low repeatability High fabrication difficulty
Fabry–Perot interferometer	Tiny size Probe design High sensitivity	High fabrication difficulty Low repeatability
Lossy mode resonance	High sensitivity	High fabrication difficulty
Surface−enhanced Raman scattering	High sensitivity High specificity	High fabrication difficulty of substrates Requires SERS labeling

## Data Availability

Datasets generated during and/or analyzed during the current study are available from the corresponding author on reasonable request.
